# Improving the Classification of PCNSL and Brain Metastases by Developing a Machine Learning Model Based on ^18^F-FDG PET

**DOI:** 10.3390/jpm13030539

**Published:** 2023-03-17

**Authors:** Can Cui, Xiaochen Yao, Lei Xu, Yuelin Chao, Yao Hu, Shuang Zhao, Yuxiao Hu, Jia Zhang

**Affiliations:** 1 Department of PET/CT Center, Jiangsu Cancer Hospital and Jiangsu Institute of Cancer Research and the Affiliated Cancer Hospital of Nanjing Medical University, Nanjing 210009, China; 2 Department of Nuclear Medicine, Nanjing First Hospital, Nanjing Medical University, Nanjing 210006, China; 3 Department of Cardiology, Nanjing First Hospital, Nanjing Medical University, Nanjing 210006, China

**Keywords:** primary central nervous system lymphoma, predictive modeling, Radiomics, machine learning, PET

## Abstract

**Background:** The characteristic magnetic resonance imaging (MRI) and the positron emission tomography (PET) findings of PCNSL often overlap with other intracranial tumors, making definitive diagnosis challenging. PCNSL typically shows iso-hypointense to grey matter on T2-weighted imaging. However, a particular part of PCNSL can demonstrate T2-weighted hyperintensity as other intracranial tumors. Moreover, normal high uptake of FDG in the basal ganglia, thalamus, and grey matter can mask underlying PCNSL in ^18^F-FDG PET. In order to promote the efficiency of diagnosis, the MRI-based or PET/CT-based radiomics models combining histograms with texture features in diagnosing glioma and brain metastases have been widely established. However, the diagnosing model for PCNSL has not been widely reported. The study was designed to investigate a machine-learning (ML) model based on multiple parameters of 2-deoxy-2-[18F]-floor-D-glucose (^18^F-FDG) PET for differential diagnosis of PCNSL and metastases in the brain. **Methods:** Patients who underwent an ^18^F-FDG PET scan with untreated PCNSL or metastases in the brain were included between May 2016 and May 2022. A total of 126 lesions from 51 patients (43 patients with untreated brain metastases and eight patients with untreated PCNSL), including 14 lesions of PCNSL, and 112 metastatic lesions in the brain, met the inclusion criteria. PCNSL or brain metastasis was confirmed after pathology or clinical history. Principal component analysis (PCA) was used to decompose the datasets. Logistic regression (LR), support vector machine (SVM), and random forest classification (RFC) models were trained by two different groups of datasets, the group of multi-class features and the group of density features, respectively. The model with the highest mean precision score was selected. The testing sets and original data were used to examine the efficacy of models separately by using the weighted average *F*1 *score* and area under the curve (AUC) of the receiver operating characteristic curve (ROC). **Results:** The multi-class features-based RFC and SVM models reached identical weighted-average *F*1 *scores* in the testing set, and the score was 0.98. The AUCs of RFC and SVM models calculated from the testing set were 1.00 equally. Evaluated by the original dataset, the RFC model based on multi-class features performs better than the SVM model, whose weighted-average *F*1 *scores* of the RFC model calculated from the original data were 0.85 with an AUC of 0.93. **Conclusions:** The ML based on multi-class features of ^18^F-FDG PET exhibited the potential to distinguish PCNSL from brain metastases. The RFC models based on multi-class features provided comparatively high efficiency in our study.

## 1. Instruction

The use of imaging techniques to assess brain lesions is crucial in diagnosing and managing neurological disorders. MRI and CT have commonly used imaging modalities, but they have limited usefulness in providing information on the metabolic activity of brain lesions. In contrast, ^18^F-FDG PET-CT is a functional imaging modality that can provide valuable information on the metabolic activity of brain lesions, particularly brain tumors.

A review of the literature suggests that 2-deoxy-2-[18F]-floor-D-glucose (^18^F-FDG) PET-CT is a valuable tool in identifying metabolically active brain tumors and monitoring treatment response [1]. Zhao et al. (2014) reported that ^18^F-FDG PET-CT had high sensitivity and specificity for detecting brain tumors and differentiating them from non-neoplastic lesions [2].

However, the accuracy and usefulness of ^18^F-FDG PET-CT in CNS diagnosis are still debated among researchers and clinicians. Some studies have reported lower accuracy rates for differentiating between benign and malignant brain tumors. The usefulness of ^18^F-FDG PET-CT may be affected by the lesion’s type and location, surrounding inflammation or edema, and the patient’s metabolic state.

Despite these limitations and controversies, the available evidence suggests that ^18^F-FDG PET-CT remains a valuable tool for assessing brain lesions, particularly in the context of brain tumors. Yang et al. (2019) [3] reported that ^18^F-FDG PET-CT and MRI had similar diagnostic accuracy in differentiating between high-grade and low-grade gliomas.

Primary central nervous system lymphoma (PCNSL) is a rare type of non-Hodgkin lymphoma that affects the brain, eyes, leptomeninges, or spinal cord. The incidence of PCNSL was 7 cases per 1,000,000 people in the USA in 2013 [4]. The PCNSL accounts for 2–3% of all brain tumors [5] (pp. 971–977). A study reported that the 2-year age-adjusted relative survival rate of PCNSL was 33%, and the corresponding 5-year survival rate of PCNSL was 26% [6]. An accurate diagnosis is crucial for the effective treatment of PCNSL. Currently, combination chemotherapy regimens that include high-dose methotrexate are considered the standard of care for newly diagnosed PCNSL [7]. In contrast, patients with brain metastases require a multidisciplinary approach that involves surgical resection, various radiation treatment modalities, cytotoxic chemotherapy, and targeted molecular treatment [8].

Neuro-imaging using cranial MRI with fluid-attenuated inversion recovery (FLAIR) and T1-weighted sequences before and after contrast injection is the preferred method for diagnosing and monitoring PCNSL [9]. However, distinguishing between PCNSL and brain metastases can be challenging since both present similar MRI signs, such as non-enhancing core and perifocal edema [10]. Moreover, a particular part of PCNSL can demonstrate T2-weighted hyperintensity as other intracranial tumors [11]. ^18^F-FDG PET can be helpful for differential diagnosis, but it has insufficient specificity [9,12].

Recent, more inspiring studies of MRI-based or PET/CT-based radiomics models combining histograms with texture features have been widely reported in diagnosing and managing glioma and metastases in the brain [13,14]. Nonetheless, due to the low morbidity of PCNSL, the relevant diagnosing model has not been widely investigated yet.

Therefore, we aim to establish several models based on ^18^F-FDG PET/CT and find an estimator with the best-predicted performance to identify PCNSL to improve diagnosis, affect patients’ management, decrease the number of indications to surgical interventions, direct the patient to the most accurate therapy, and, therefore, affect their quality of life.

## 2. Materials and Methods

Our study follows the guideline, transparent reporting of a multivariable prediction model for individual prognosis or diagnosis (TRIPOD) [15]. The statement adhered to the supplement materials as a part of the study (Table S1).

### 2.1. Study Participants

**Figure 1:**
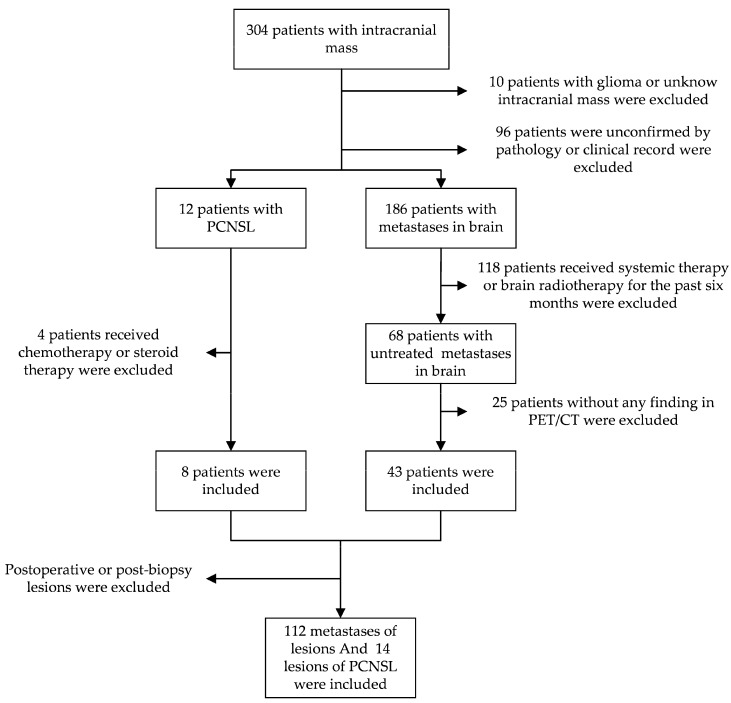
The pathway of inclusion criteria for patients and lesions. One hundred twenty-six lesions were included in the total.

The study retrospectively reviewed patients with intracranial mass who received an ^18^F-FDG PET/CT at Jiangsu Cancer Hospital from May 2016 to May 2022. Patients with PCNSL confirmed by pathology and brain metastases confirmed by pathology or clinical history without receiving systemic therapy or brain radiotherapy for the past six months. Due to patients’ compliance, the biopsy of brain metastases cannot be feasible for all the patients whose primary tumor was pathologically confirmed. All the lesions were not postoperative or post-biopsy (Figure 1).

### 2.2. ^18^F-FDG PET/CT Protocol

^18^F-FDG PET/CT protocol followed the European Association of Nuclear Medicine’s guidelines [16]. Patients fasted for at least 6 h. The plasma glucose level of all the patients was in a range from 4.0 mmol/L to 8.3 mmol/L. For patients with diabetes, additional restrictions were applied. Only intermediate-acting or short-acting insulin was allowed within 12 h before the administration of ^18^F-FDG, and the application of metformin was compromised. The radioactivity of ^18^F-FDG for intravenous injection was calculated by body weight, 4.1 ± 0.82 Mbq/kg (range from 2.96 MBq/kg to 5.55 Mbq/kg). The acquisition of the brain starts at 77 ± 2.9 min (range from 74 to 82 min) after ^18^F-FDG injection when the PET scan of the torso (from the canthus line to the thigh) was completed.

The brain scan is a separate procedure. The PET/CT (Discover 710 STD GE Healthcare, Waukesha, WI, USA) image acquisition consisted of a 10-min emission scanning with one bed for the brain and low-dose CT for attenuation correction. The voxel size was 3.65 × 3.65 × 3.75 in mm with a matrix of 192 × 192. The reconstruction is Vue Point FX with 24 subsets and 2-times iterations. Low-dose CT used 3.75 mm slice thickness, pitch 1.375:1, 140 kV with Auto-mA.

### 2.3. Segmentation of Images

All the PET/CT images, relevant MRI, and related contrast-enhanced CT images were reviewed using PET VCAR with Integrated Registration, a component of the Advantage Workstation (version 4.6, GE Healthcare, Waukesha, WI, USA).

Segmentation of lesions was performed by two clinical radiologists with over five years of experience. The volume of interest (VOI) was checked by radiology and nuclear medicine physicians with a career in oncological PET/CT interpretation over ten years.

Segmentation of PET volumes was based on the iterative image thresholding method (ITM), which yielded reliable PET volume estimation as previously reported [17]. Relevant MRI and contrast-enhanced CT were used as the reference to adjust the edge of VOIs manually. VOIs were saved and exported as the radiotherapy structure set (RTSS).

### 2.4. Feature Extraction

All the characters were divided into two groups, the group of density features and the group of multi-class features (Table S2). Briefly, the density-features group contains 10% percentile, 90% percentile, energy, maximum, minimum, and range. The multi-classes-features group includes all first-order characters and the texture characters, such as the gray-level co-occurrence matrix (GLCM), modification of grey-level difference matrix (GLDM), gray-level run length matrix (GLRLM), gray-level size zone matrix (GLSZM), and neighboring gray-tone difference matrix (NGTDM), 93 features in total.

As the unit of the pixel value is Becquerel per mL, PET images were normalized by the SUV factor Formula (1) and resampled to a uniform voxel size of 2 × 2 × 2 mm^3^. PyRadiomics (V3.01) (https://pyradiomics.readthedocs.io/en/latest/index.html, accessed on 3 May 2022) was used to extract all features [18]. The bin width of 0.5 was derived by dividing the maximum range by 64 [19].

(1) $$SUVfactor=\frac{W}{D\times 2^{\left(-t/T\right)}}$$

Formula (1). *W*: Body weight (g), *D*: Injection dose (Bq), *t*: Delay between injection time and scan time (s), *T*: Half-life of the isotope (s).

### 2.5. Model Training and Validation

#### 2.5.1. Statistical Analysis

**Figure 2:**
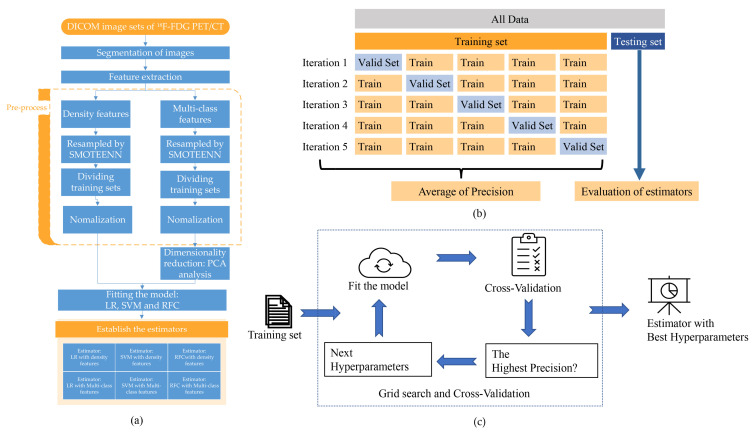
Fit the model and internal cross-validation. (**a**) The schemes of establishing the estimators. (**b**) The datasets were split into the training set and the testing set. The training set was divided into five folds for training and cross-validation. (**c**) The hyperparameters with the highest precision score in cross-validation were chosen.

The present study employed a statistical analysis of three primary steps: resampling, dimensionality reduction, and estimator establishment (Figure 2a). Specifically, to address the issue of imbalanced datasets, the researchers utilized the synthetic minority over-sampling technique and edited nearest neighbors (SMOTEENN) algorithm. SMOTEENN is a hybrid approach that combines the synthetic minority over-sampling technique (SMOTE) and edited nearest neighbors (ENN) algorithms. SMOTE generates synthetic minority class samples to balance the class distribution, while ENN removes examples considered noisy or belonging to the majority class. By combining these two techniques, SMOTEENN can oversample minority class examples and remove potentially noisy or irrelevant examples from the dataset. The tools were provided by mbalanced-learn (Version: 0.9.1) (https://imbalanced-learn.org/stable/, accessed on 18 May 2022) 

Principal component analysis (PCA), a linear method known for reducing the dimensions of a dataset while retaining the most relevant information, was employed to achieve the aim mentioned above. The PCA was achieved by transforming the original n-dimensional dataset into a new dataset using an orthogonal transformation [14]. For the last step, three classification algorithms were selected: support vector machine (SVM), logistic regression (LR), and random forest classification (RFC). SVM is a particularly effective classifier for small machine-learning tasks [20]. The LR classifier, while running faster, places greater emphasis on feature engineering [21]. On the other hand, RFC is known to reduce overfitting by averaging decision trees, making it a relatively stable classification method. However, it requires more time to train the model due to its complex calculation process [22]. All the tools above were provided by the scilearn-kit (Version: scikit-learn 1.1.2) (https://scikit-learn.org/stable/ accessed on 6 August 2022).

#### 2.5.2. Pre-Process of Datasets

Two groups of original datasets were separately resampled by imbalanced-learn (Version: 0.9.1) (https://imbalanced-learn.org/stable/, accessed on 18 May 2022). The method of SMOTEENN was used to balance the datasets [23,24].

Two datasets were divided into training sets and testing sets with a ratio of 2:1 using the scilearn-kit (Version: scikit-learn 1.1.2) (https://scikit-learn.org/stable/, accessed on 6 August 2022. All of the data were normalized by Standard-Scaler provided by scilearn-kit.

#### 2.5.3. Dimensionality Reduction

Principal component analysis (PCA) was used for dimensionality reduction for the multi-class features group. PCA reduces high-dimensional features into a small number of principal components (PCs). The PCs will be retained until the cumulative-explained variance is over 0.9.

The dimension of the density-features group was not reduced. Because only six dimensionalities exist in the datasets, dimensionality reduction is unnecessary.

#### 2.5.4. Fitting the Model and Internal Cross-Validation

Two groups of data were fitted to logistic regression (LR), support vector machine (SVM), and random forest classification (RFC) models.

Hyperparameters were determined by grid search with five-fold cross-validation (Figure 2b,c) [25,26]. Briefly, the dataset was split into five folds. In the initial iteration, the first fold was used to validate the model, and the rest folds were used for the training of the model. In the second iteration, the second fold is used as the validation set, while the rest is the training set. This process was repeated five times. The precision Formula (2) of each iteration was averaged. All of the hyperparameters were traversed by grid search. The hyperparameters of each model with the highest precision were selected. Finally, trained by training sets with the best hyperparameters, the six estimators were established from three different models with two data sets.

#### 2.5.5. Evaluation of Estimators

The testing sets and original datasets (the dataset without resampling) were used to evaluate the estimator.

The receiver operating characteristic curve (ROC) with the area under the curve (AUC) is presented. The *F*1 *score* Formula (2) is a machine-learning metric used in classification models [27]. For imbalanced data, we use the weighted average *F*1 *score* to compare the efficiency of the estimators.

(2) $$Precision=\frac{True\;positives}{True\;Positives+False\;positives}$$

(3) $$Recall=\frac{True\;positives}{True\;positives+False\;Nagetives}$$

(4) $$F1\;score=2\times \frac{Precision\cdot Recall}{Precison+Recall}$$

Formula (2). The definition of precision (2), recall (3), and Average *F*1 *score* (4).

## 3. Result

### 3.1. Study Participants

The characteristics of patients are demonstrated in Table 1. In total, 8 patients with PCNSL and 43 patients with metastases in the brain were included, with 14 lesions of PCNSL and 112 lesions of metastases in the brain (Figure 1). The primary tumor of all the brain metastases patients was pathologically confirmed. One of the patients, whose primary tumor was adenocarcinoma of the lung, underwent a craniotomy biopsy. Finally, the brain metastases of the lung carcinoma were confirmed. The pathology result of all patients with PCNSL was confirmed by stereotaxic needle biopsy. There is no significant difference in sex and age. The SUVmax of PCNSL and metastases is significantly different.

### 3.2. Dimensionality Reduction

**Figure 3:**
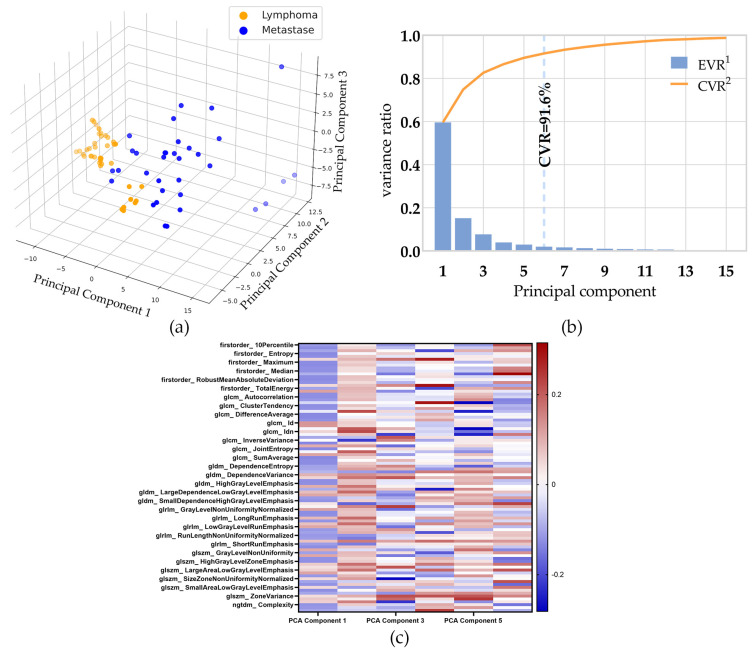
The result PCA. (**a**) The data of the first three principal components in the multi-class features group training set. (**b**) The individual-explained variance ratio and cumulative-explained variance ratio for each principal component. (**c**) The heat map shows a matrix of the PCA loading vectors.^1^ EVR: individual-explained variance ratio. ^2^ CVR: cumulative-explained variance ratio.

The study used PCA to project 93 features in the multi-classes-features group to six dimensions. The data of the first three principal components in the training set of the multi-class-features group is shown in Figure 3a. The individual-explained variance ratio and cumulative-explained variance ratio for each principal component are shown in Figure 3b. The cumulative-explained variance ratio of the third principal component is 82.6%, and the sixth is 91.6%, meaning the first 6 principal components contained 91.6% of the information of all 93 features.

The PCA loading vectors are shown in Figure 2c and Supplement Table S3. The multi-features dataset was converted from its original dimension to the reduced PCA dimension by using the vectors in the linear transformation.

### 3.3. Modeling and Validating

#### 3.3.1. Fit the Model and Internal Cross-Validation

The hyperparameters of all the estimators are shown in Table 2.

**Figure 4:**
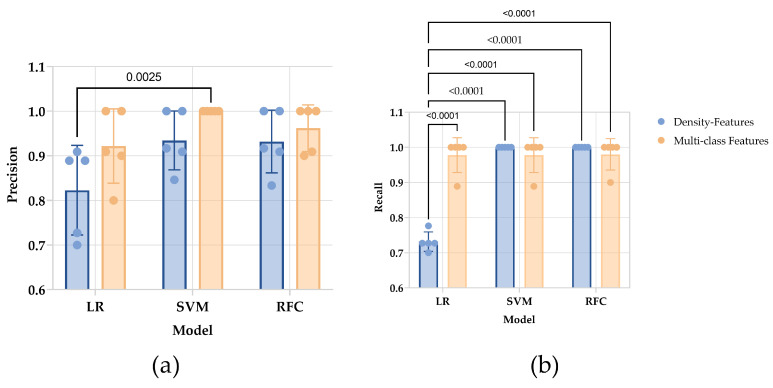
The result of internal cross-validation of chosen estimators. (**a**) For the precision of each estimator, a significant difference was observed in the SVM model with multi-class features and the LR model with density features. (**b**) The recall of each estimator. The LR model with density shows the lowest recall. The ANOVA analysis with multiple comparisons calculated *p*-value.

The precision between different models (*p* = 0.0137) and between datasets (*p* = 0.0174) are discrepant. In multiple comparisons between values of precision, only the difference between the SVM model trained by multi-class features and the LR model trained by density features is observed (*p* = 0.0025). The recall of the LR model trained by density features is lower than the others (*p* < 0.0001), while there is no difference was found between the others (Figure 4).

#### 3.3.2. Evaluation of Estimators

**Figure 5:**
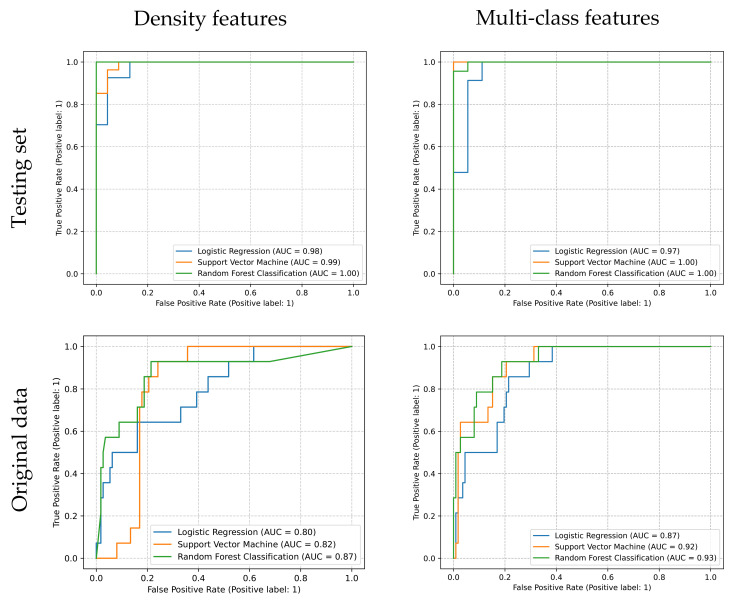
The ROC of all estimators. Tested by original data, the AUC of SVM and RFC trained by multi-class features are 0.92 and 0.93.

The weighted average *F*1 *score* of estimators is shown in Table 3. Although all the ROC of estimators shows a nearly perfect performance in the testing set, only SVM and RFC trained by multi-class features exhibit acceptable results, of which the AUCs are 0.92 and 0.93 (>0.9) (Figure 5).

## 4. Discussion

The study established a model to classify PCNSL and neuro-metastases, combining histogram and high-order characteristics from lesions in ^18^F-FDG PET images. The technique, dimensionality reduction and the balance of data sets, was adopted to reduce the possibility of overfitting.

The SVM and RFC models trained by the multi-class features data set and the RFC models trained by density features show the highest *F*1 *scores* and AUCs validated by the testing set. However, evaluated by the data sets without resampling, the *F*1 *scores* and AUCs’ reduction of all six estimators can be observed. Nevertheless, the *F*1 *score* and AUC of the RFC models trained by the multi-class features were still acceptable and relatively higher than others evaluated by the testing and original data set.

^18^F-FDG PET-CT is a sensitive screening tool for PCNSL patients suspected of systemic involvement [7]. However, A low diagnostic yield of PCNSL for initial staging has been reported [28]. Even if the limitation of ^18^F-FDG PET in neuro-oncology is widely accepted, some studies argued that the different SUVmax and tumor-normal ratios could be observed in PCNSL and metastases in the brain [12,29]. A similar result can also be drawn from our data; sensitivity and specificity are 71.43% and 73.21%, with a cut-off of 14.42. However, the change SUVmax and tumor-normal ratios may not be conspicuous in atypical PCNSL [30]. Precisely as we noticed, some lesions of PCNSL can be concealed by the high metabolism of the cerebral cortex. In recent years, ^18^F-FDG PET or MRI-based radiomics features have been reported to distinguish the PCNSL and glioblastoma, which provides a reliable noninvasive method [31,32,33,34]. The multi-feature-based diagnosing method should potentially promote the performance in the differential diagnosis between PCNSL and brain metastases. It is just what we discussed in our study to establish a method based on radiomics to increase the diagnosis accuracy of the PCNSL and brain metastases interpreted from ^18^F-FDG PET.

Due to the disparate incidence of PCNSL and brain metastases, the data set can be highly unbalanced. The incidence of PCNSL was 7 cases per 1,000,000 people in the USA in 2013 [4]. The PCNSL accounts for 2–3% of all brain tumors [5] (pp. 971–977). Relatively, brain metastases develop in approximately 10% to 30% of adults and 6% to 10% of children with cancer [35]. Training with unbalanced datasets may lead to overfitting and underfitting. The synthetic minority over-sampling technique (SMOTE) can be an appropriate option for dealing with imbalanced datasets [24]. The SMOTE is a way to deal with the minority classes in a dataset. This algorithm’s fundamental idea is to analyze, simulate, and add the new sample simulated artificially into the original dataset to balance the classes in the original data. In our study, the hyper-sampling method was used. The method combines SOMTE with edited nearest neighbors (ENN), an under-sampling technique that removes the majority class to match the minority class [36]. The method has been used in several clinical studies [37,38,39,40].

Actually, for the sure size of the training set, the predictive performance of models decreases with increasing dimensionality [41]. The six visually recognizable features were defined as the group of density. Ninety-three features in the multi-class features group were extracted for PET imaging. The multi-class features can be redundant, and some features can be highly related, which may lead to the over-fitting of the models. It is vital to reduce dimensionality without losing information. PCA determines a set of orthogonal vectors called principal components, defined by a linear combination of the original variables and ordered by the amount of variance explained in component directions [42]. The cumulative-explained variance ratio, the summary of explained variance ratio, has been set to 0.9, which means more than 90 percent of variation from the 93 features has been retained.

In our study, besides the AUCs of ROCs, the weight-average *F*1 *scores* were used to evaluate the predicted performance of estimators. While ROC was unaffected by skew, precision–recall curves suggest that ROC may mask poor performance [43]. The weight-average *F*1 *score* is the harmonic mean of precision (also called positive predictive value) and recall (indicated the sensitivity), widely used in information retrieval and information extraction evaluation [44]. In our study, the weighted average *F*1 *scores* were used to evaluate the performance of estimators, which calculates the weighted mean of all per-class *F*1 *scores* while considering each class’s support, eliminating the effect of unbalanced data sets.

For the five sixths estimators, the *F*1 *scores* resulting from the testing set are more prominent than 0.9. The result indicated that precision and sensitivity could be excellent in the testing set. ROC and AUC can also display similar results. In order to evaluate the predicted performance in the real world and the generalization ability of the estimators, we used the original data sets (without resampling) to re-evaluate all estimators. We noticed that all estimators’ *F*1 *scores* or AUCs have a decrease in a certain degree tested by original data sets (without resampling) while considering the class imbalance. Especially in the RFC model trained by density features, the *F*1 *score* is 1.00 in the testing set and decreases to 0.82 in the original data. We conjecture that overfitting this estimator may decrease the estimators’ performance, as reported [45].On the other hand, the estimator generated from the RFC model trained by multi-class features performs well for both the testing data set and the original data set (without resampling). We conjecture that the characters of the random forests algorithm decrease the possibility of overfitting. Because the random forests deal with the problem of overfitting by creating multiple trees, with each tree trained slightly differently, it overfits differently. The sufficient diagnostic information provided by the multi-class features and the combination of each decision tree offset the effect of overfitting each decision tree.

The low incidence of PCNSL and restricted enrollment criteria restrict the sample size, and further multicenter studies are urgently required. The utilization of various machine learning algorithms has significantly enhanced the efficacy of identifying primary central nervous system lymphoma (PCNSL) and brain metastases. However, it has concurrently augmented the complexity of the practical implementation of these techniques in clinical settings. The random forest model exhibits superior accuracy when dealing with high-dimensional data. Nevertheless, the random forest model’s interpretability is greatly diminished by the utilization of multiple decision tree models to determine the final classification outcome through voting.

The ML model based on ^18^F-FDG can improve the diagnosis of brain lesions by providing clinicians with more precise and consistent information, which can lead to faster and more effective treatment decisions. Radiomics models, which use AI algorithms to analyze medical images, have shown promise in differentiating between brain lesions, including PCNSL and brain metastases.

However, while The ML model based on ^18^F-FDG has shown potential in improving the diagnosis of brain lesions, more research is needed to fully understand their clinical impact and how to integrate them into clinical practice. Clinicians must be aware of these tools’ limitations and potential biases and ensure their use is evidence-based and clinically relevant.

## 5. Conclusions

The SUVmax of ^18^F-FDG PET is a proven semi-quantitative indicator; the combination of radiomics and machine learning promotes the performance of PCNLS and brain metastases diagnosis. The *F*1 *score* and AUC of the RFC model trained by multi-class features are 0.85 and 0.93. The RFC model trained by multi-class features has the potential to revolutionize brain lesions diagnosis and improve patient outcomes. However, they need to integrate into clinical practice cautiously and consider their limitations and biases. More research is needed to fully understand the clinical impact of the model and how it can be best utilized in clinical settings.

## Figures and Tables

**Table 1:** Characteristics of patients.

Characteristics	PCNSL	Metastases	*p* Value
Sex			0.0986 ^2^
Male	3	31	
Female	5	12	
Age	56.00 ± 13.98	59.49 ± 11.74	0.4570 ^3^
SUVmax ^1^	20.14 ± 7.58	12.80 ± 4.84	0.0006 ^3^
Pathology			
B cell lymphoma	8		
Squamous carcinoma ^4^		12	
Adenocarcinoma ^4^		22	
Melanoma ^4^		3	
Renal clear cell cancer ^4^		2	
Neuroendocrine carcinoma ^4^		2	

^1^ The AUC of ROC is 0.78. If the cut-off of SUVmax is 14.42, the sensitivity and specificity are 71.43% and 73.21%, and the *F*1 *score* is 0.287. ^2^ The statistical method is Fisher’s exact test. ^3^ The statistical method is the *t*-test. ^4^ No difference in the average SUVmax among different pathological types was found (*p* = 0.5213) (Figure S1).

**Table 2:** Hyperparameters and precision of estimators.

	Density Features		Multi-Class Features	
	Hyperparameters	Precision	Hyperparameters	Precision
LR	C: 1.0 dual: True multi_class: ovr penalty: l2 solver: liblinear	0.822 ± 0.090	C: 1.4 dual: False multi_class: ovr penalty: l1 solver: liblinear	0.921 ± 0.074
SVM	C: 2.81 gamma: 2.21 kernel: rbf	0.934 ± 0.060	C: 7.01 gamma: 0.21 kernel: poly	1.0 ± 0.0
RFC	bootstrap: False max_depth: 20 max_features: log2 min_samples_leaf: 4 min_samples_split: 16 n_estimators: 500	0.932 ± 0.063	bootstrap: False max_depth: 5 max_features: sqrt min_samples_leaf: 2 min_samples_split: 8 n_estimators: 500.	0.962 ± 0.047

**Table 3:** The weighted average *F*1 *scores* of estimators.

	Density Features		Multi-Class Features	
	Testing Set	Original Data	Testing Set	Original Data
LR	0.86	0.79	0.93	0.82
SVM	0.96	0.78	0.98	0.83
RFC	1.00	0.82	0.98	0.85

## Data Availability

The data are not publicly available due to institutional data sharing restrictions.

## Supplementary Materials

The following supporting information can be downloaded at: https://www.mdpi.com/article/10.3390/jpm13030539/s1, Figure S1: The SUVmax of brain metastases with different pathology; Table S1: The TRIPOD checklist, Table S2: The features list of the group of density characters and the group of multi-class characters; Table S3: The PCA loading vectors.
