# Supplementary material for: Improving the Classification of PCNSL and Brain Metastases by Developing a Machine Learning Model Based on 18F-FDG PET
Source: J Pers Med. 2023 Mar 17;13(3):539. doi: 10.3390/jpm13030539 (PMC10056979; doi:10.3390/jpm13030539)
Supplement: Supplementary file 1 [file jpm-13-00539-s001.zip › jpm-2190103-supplementary.pdf]

Supplementary Material

Table S1. The TRIPOD checklist

Section/topic	Item	Development or validation?	Checklist item	explanation
Title and abstract				
Title	1	D;V	Identify the study as developing and/or validating a multivariable prediction model, the target population, and the outcome to be predicted	Described in Title
Abstract	2	D;V	Provide a summary of objectives, study design, setting, participants, sample size, predictors, outcome, statistical analysis, results, and conclusions	Presented in Abstract
Introduction				
Background and objectives	3a	D;V	Explain the medical context (including whether diagnostic or prognostic) and rationale for developing or validating the multivariable prediction model, including references to existing models	Presented in Introduction
	3b	D;V	Specify the objectives, including whether the study describes the development or validation of the model, or both	Develop a classification model.
Methods				
Source of data	4a	D;V	Describe the study design or source of data (for example, randomised trial, cohort, or registry data), separately for the development and validation data sets, if applicable	This is a retrospective study.<br>Details in the section of study participants
	4b	D;V	Specify the key study dates, including start of accrual; end of accrual; and, if applicable, end of follow-up	May 2016 to May 2022
Participants	5a	D;V	Specify key elements of the study setting (for example, primary care, secondary care, general population) including number and location of centres	This is a single-center study
	5b	D;V	Describe eligibility criteria for participants	Details in the section of study participants
	5c	D;V	Give details of treatments received, if relevant	Patients who received treatment were excluded.

Outcome	6a	D;V	Clearly define the outcome that is predicted by the prediction model, including how and when assessed	This is a classification model.
	6b	D;V	Report any actions to blind assessment of the outcome to be predicted	Predict the type of pathology which is blind to images.
Predictors	7a	D;V	Clearly define all predictors used in developing the multivariable prediction model, including how and when they were measured	All the information is presented in the segmentation of images, Feature extraction.
	7b	D;V	Report any actions to blind assessment of predictors for the outcome and other predictors	The VOI blinded to the classification result
Sample size	8	D;V	Explain how the study size was arrived at.	All patients who met the inclusion criteria were included
Missing data	9	D;V	Describe how missing data were handled (for example, complete-case analysis, single imputation, multiple imputation) with details of any imputation method	None of the data was missing
Statistical analysis methods	10a	D	Describe how predictors were handled in the analyses	Described in pre-process of datasets and dimensionality reduction
	10b	D	Specify type of model, all model-building procedures (including any predictor selection), and method for internal validation	Described in fitting the model and internal cross-validation
	10c	V	For validation, describe how the predictions were calculated	As described in the External test
	10d	D;V	Specify all measures used to assess model performance and, if relevant, to compare multiple models	The weighted average F1 score and AUC of ROC
	10e	V	Describe any model updating (for example, recalibration) arising from the validation, if done	NOT APPLICABLE
Risk groups	11	D;V	Provide details on how risk groups were created, if done	NOT INCLUDED
Development v validation	12	V	For validation, identify any differences from the development data in setting, eligibility criteria, outcome, and predictors	
Results				
Participants	13a	D;V	Describe the flow of participants through the study, including the number of participants with and without the outcome and, if applicable, a summary of the follow-up time. A diagram may be helpful	Presented in Figure 1 and Table 1.

	13b	D;V	Describe the characteristics of the participants (basic demographics, clinical features, available predictors), including the number of participants with missing data for predictors and outcome	Presented in the study participants and Table 1.
	13c	V	For validation, show a comparison with the development data of the distribution of important variables (demographics, predictors and outcome).	
Model development	14a	D	Specify the number of participants and outcome events in each analysis	Presented in Table 2
	14b	D	If done, report the unadjusted association between each candidate predictor and outcome	This has not been performed.
Model specification	15a	D	Present the full prediction model to allow predictions for individuals (that is, all regression coefficients, and model intercept or baseline survival at a given time point)	The features and hyperparameters used to create the model are presented in the results section.
	15b	D	Explain how to use the prediction model	
Model performance	16	D;V	Report performance measures (with CIs) for the prediction model	The ROC and the F1 score
Model updating	17	V	If done, report the results from any model updating (that is, model specification, model performance)	
Discussion				
Limitations	18	D;V	Discuss any limitations of the study (such as nonrepresentative sample, few events per predictor, missing data)	The limitation is illustrated in the discussion section.
Interpretation	19a	V	For validation, discuss the results with reference to performance in the development data, and any other validation data	Validated by data without resampling.
	19b	D;V	Give an overall interpretation of the results, considering objectives, limitations, results from similar studies, and other relevant evidence	In discussion section
Implications	20	D;V	Discuss the potential clinical use of the model and implications for future research	
Other information				
Supplementary information	21	D;V	Provide information about the availability of supplementary resources, such as study protocol, Web calculator, and data sets	Table S2 provides all the features used in the study （Table S2）.

Funding	22	D;V	Give the source of funding and the role of the funders for the present study	In Declaration.

*Items relevant only to the development of a prediction model are denoted by D, items relating solely to a validation of a prediction model are denoted by V, and items relating to both are denoted D;V.

Table S2. The features list of the group of density characters and the group of multi-class characters

	Group of Multi-Class features	Group of density features
**Feature Class**	Feature Name	Feature Name
**firstorder**	10Percentile	10Percentile
	90Percentile	90Percentile
	Energy	Maximum
	Entropy	Minimum
	InterquartileRange	Range
	Kurtosis	Energy
	Maximum	
	MeanAbsoluteDeviation	
	Mean	
	Median	
	Minimum	
	Range	
	RobustMeanAbsoluteDeviation	
	RootMeanSquared	
	Skewness	
	TotalEnergy	
	Uniformity	
	Variance	
**glcm**	Autocorrelation	
	ClusterProminence	
	ClusterShade	
	ClusterTendency	
	Contrast	
	Correlation	
	DifferenceAverage	
	DifferenceEntropy	
	DifferenceVariance	
	Id	
	Idm	
	Idmn	
	Idn	
	Imc1	
	Imc2	
	InverseVariance	
	JointAverage	
	JointEnergy	
	JointEntropy	
	MCC	

	MaximumProbability
	SumAverage
	SumEntropy
	SumSquares
	DependenceEntropy
	DependenceNonUniformity
	DependenceNonUniformityNormalized
	DependenceVariance
	GrayLevelNonUniformity
	GrayLevelVariance
	HighGrayLevelEmphasis
	LargeDependenceEmphasis
	LargeDependenceHighGrayLevelEmphasis
	LargeDependenceLowGrayLevelEmphasis
	LowGrayLevelEmphasis
	SmallDependenceEmphasis
	SmallDependenceHighGrayLevelEmphasis
	SmallDependenceLowGrayLevelEmphasis
	GrayLevelNonUniformity
	GrayLevelNonUniformityNormalized
	GrayLevelVariance
	HighGrayLevelRunEmphasis
	LongRunEmphasis
	LongRunHighGrayLevelEmphasis
	LongRunLowGrayLevelEmphasis
	LowGrayLevelRunEmphasis
	RunEntropy
	RunLengthNonUniformity
	RunLengthNonUniformityNormalized
	RunPercentage
	RunVariance
	ShortRunEmphasis
	ShortRunHighGrayLevelEmphasis
	ShortRunLowGrayLevelEmphasis
**glszm**	GrayLevelNonUniformity
	GrayLevelNonUniformityNormalized
	GrayLevelVariance
	HighGrayLevelZoneEmphasis
	LargeAreaEmphasis
	LargeAreaHighGrayLevelEmphasis
	LargeAreaLowGrayLevelEmphasis
	LowGrayLevelZoneEmphasis
	SizeZoneNonUniformity

	SizeZoneNonUniformityNormalized
	SmallAreaEmphasis
	SmallAreaHighGrayLevelEmphasis
	SmallAreaLowGrayLevelEmphasis
	ZoneEntropy
	ZonePercentage
	ZoneVariance
**ngtdm**	Busyness
	Coarseness
	Complexity
	Contrast
	Strength

Table S3. The PCA loading vectors

Features		PCA Component 1	PCA Component 2	PCA Component 3	PCA Component 4	PCA Component 5	PCA Component 6
firstorder	10Percentile	-0.112817629	0.025943668	-0.113812421	0.013057658	-0.108794692	0.214837365
firstorder	90Percentile	-0.125775099	0.048282253	-0.072328859	0.010512173	-0.059666243	0.10668376
firstorder	Energy	-0.100829476	0.094310343	0.007789189	-0.199713764	0.098378732	0.139199344
firstorder	Entropy	-0.131019331	-0.009438704	0.059701806	0.019628285	-0.046500535	0.01809269
firstorder	InterquartileRange	-0.12744646	0.07013338	-0.027814876	-0.019731245	0.026338829	-0.01475991
firstorder	Kurtosis	0.049331837	0.090031753	0.17595536	0.276503475	0.023156254	0.061558916
firstorder	Maximum	-0.126572607	0.057493535	-0.049687659	0.040038992	-0.081886374	0.067842814
firstorder	MeanAbsoluteDeviation	-0.128412207	0.068978246	-0.02561583	0.006317289	0.000759849	-0.01564571
firstorder	Mean	-0.122484544	0.041432331	-0.087302266	-0.003430344	-0.067273756	0.154624778
firstorder	Median	-0.122282199	0.041333134	-0.087208981	-0.02207969	-0.054835001	0.166091581
firstorder	Minimum	-0.08831641	-0.004977091	-0.146422049	0.028966283	-0.165211309	0.314773624
firstorder	Range	-0.127172905	0.075096444	-0.006974784	0.039833439	-0.040739537	-0.03461616
firstorder	RobustMeanAbsoluteDeviation	-0.127967667	0.069001861	-0.027847467	-0.0101033	0.019556166	-0.01385084
firstorder	RootMeanSquared	-0.123299419	0.043388232	-0.084189066	-0.002459031	-0.063762168	0.145531787
firstorder	Skewness	0.044803346	0.077693653	0.145766869	0.294122452	-0.105694681	-0.095986679
firstorder	TotalEnergy	-0.100829476	0.094310343	0.007789189	-0.199713764	0.098378732	0.139199344

firstorder	Uniformity	0.116599642	0.086737038	-0.128682386	-0.014593528	0.03812797	-0.028559708
firstorder	Variance	-0.125083128	0.077757441	-0.036061204	-0.009985811	0.079866582	-0.068768966
18							
glcm	Autocorrelation	-0.125309666	0.073931329	-0.0287298	-0.037549332	0.101580733	-0.049534598
glcm	ClusterProminence	-0.115236366	0.086030843	-0.031528357	-0.034045961	0.142355488	-0.138908184
glcm	ClusterShade	-0.044626371	-0.006901487	-0.006256869	0.292514269	-0.281211005	-0.130367215
glcm	ClusterTendency	-0.126121719	0.073510044	-0.024171252	-0.005414662	0.066555905	-0.083876928
glcm	Contrast	-0.122371449	0.038136964	-0.061474825	0.120845823	0.027930762	-0.097060293
glcm	Correlation	-0.024616313	0.221271753	0.047557197	-0.051682993	-0.246399368	-0.075811338
glcm	DifferenceAverage	-0.130181342	0.002186167	-0.038341027	0.0936345	-0.007639803	-0.015204748
glcm	DifferenceEntropy	-0.130676782	-0.017505369	0.02868243	0.062013636	-0.056335938	0.009941397
glcm	DifferenceVariance	-0.123441774	0.043267229	-0.050591116	0.113599643	0.021512541	-0.110988583
glcm	Id	0.1280512	0.060257939	-0.002496651	-0.058659534	0.034637813	-0.057672169
glcm	Idm	0.128587268	0.052079758	0.004193615	-0.062449936	0.03965773	-0.062762816
glcm	Idmn	-0.008956282	0.206775924	0.097439661	-0.040265063	-0.278371635	0.009846512
glcm	Idn	0.052079812	0.222525533	0.036329434	-0.053978616	-0.154636366	-0.071446881
glcm	Imc1	-0.011036791	0.070424696	-0.213697017	-0.03514034	-0.245675692	0.165769324
glcm	Imc2	-0.097442	-0.072417755	0.205379325	0.036773651	0.025912761	-0.063781821
glcm	InverseVariance	0.004772159	-0.21603094	0.155264573	-0.098470901	0.026783996	0.040536708

glcm	JointAverage	-0.131538062	0.047491693	-0.007993595	-0.01333747	0.025064206	-0.002901549
glcm	JointEnergy	0.101476599	0.122400452	-0.162670169	0.004999784	0.050357426	-0.01097908
glcm	JointEntropy	-0.130262599	-0.003983087	0.054365346	0.023013027	-0.080055089	0.007158888
glcm	MCC	-0.011283716	0.197158627	-0.025344364	-0.072128387	-0.125595108	-0.015328159
glcm	MaximumProbability	0.113069359	0.101558069	-0.111755256	0.006081462	0.078357316	-0.026986498
glcm	SumAverage	-0.131538062	0.047491693	-0.007993595	-0.01333747	0.025064206	-0.00290155
glcm	SumEntropy	-0.127795444	-0.003153894	0.088808026	0.007439874	-0.085564395	-0.011724768
glcm	SumSquares	-0.126877325	0.067675876	-0.031504785	0.018394003	0.060026124	-0.087343516
gldm	DependenceEntropy	-0.102971557	0.089251368	0.14797567	-0.061410317	-0.17696039	-0.048507319
gldm	DependenceNonUniformity	-0.100088761	0.12172558	0.089633187	-0.115851818	0.101003725	0.108338376
gldm	DependenceNonUniformityNormalized	-0.081210401	-0.108397154	-0.135594288	0.159817005	0.171298832	0.120382133
gldm	DependenceVariance	0.09320135	0.14050296	0.111173527	0.006032334	0.049559576	-0.00946956
gldm	GrayLevelNonUniformity	0.053000387	0.169710415	0.205613702	0.087758008	0.089896999	0.153970678
gldm	GrayLevelVariance	-0.126066504	0.071625425	-0.029643902	-0.011913782	0.084376503	-0.070998117
gldm	HighGrayLevelEmphasis	-0.125027917	0.075008303	-0.025616102	-0.044385325	0.103522618	-0.050867455
gldm	LargeDependenceEmphasis	0.100009541	0.16977572	0.016291332	0.036210975	0.009333298	-0.002189223
gldm	LargeDependenceHighGrayLevelEmphasis	-0.090915688	0.105556428	0.06854451	-0.249554924	0.14634198	0.00735759
gldm	LargeDependenceLowGrayLevelEmphasis	0.080232733	0.182788471	-0.105641026	0.109327199	0.035587013	0.077484229
gldm	LowGrayLevelEmphasis	0.107575929	0.089196856	-0.141900928	0.022193193	0.112626707	-0.05970987

gldm	SmallDependenceEmphasis	-0.111977647	-0.051563401	-0.09582126	0.117779101	0.080167655	0.122952906
gldm	SmallDependenceHighGrayLevelEmphasis	-0.119219626	0.060393457	-0.060237728	0.075186643	0.097385638	-0.136508953
gldm	SmallDependenceLowGrayLevelEmphasis	0.03108347	-0.174061154	-0.092639425	0.027388011	0.158283358	0.232869896
glrlm	GrayLevelNonUniformity	0.014595144	0.14934932	0.239990125	-0.112636834	-0.042313624	0.104820171
glrlm	GrayLevelNonUniformityNormalized	0.112873167	0.081106692	-0.158232055	-0.04056602	0.013055707	-0.033521244
glrlm	GrayLevelVariance	-0.126140038	0.071552818	-0.027906439	-0.004552302	0.081302873	-0.078264651
glrlm	HighGrayLevelRunEmphasis	-0.125480332	0.074308667	-0.025365386	-0.033506541	0.097990809	-0.057862367
glrlm	LongRunEmphasis	0.086980808	0.183411604	0.003879245	0.122035657	0.06371106	0.088664208
glrlm	LongRunHighGrayLevelEmphasis	-0.118577872	0.094991905	0.012544465	-0.101165968	0.117897798	-0.042903384
glrlm	LongRunLowGrayLevelEmphasis	0.07614801	0.180207712	-0.081889156	0.147758963	0.076007544	0.130558714
glrlm	LowGrayLevelRunEmphasis	0.108406272	0.078794178	-0.155644129	-0.014813334	0.091748773	-0.075727468
glrlm	RunEntropy	-0.11393373	0.086214558	0.131043872	0.024769574	-0.089106373	-0.00265329
glrlm	RunLengthNonUniformity	-0.104127143	0.097612772	0.074052379	-0.192327176	0.046322725	0.069762037
glrlm	RunLengthNonUniformityNormalized	-0.114213108	-0.119874024	-0.052264366	0.044860657	0.026265587	0.076021345
glrlm	RunPercentage	-0.109139786	-0.145311571	-0.03784403	0.01583247	0.013663138	0.050294832
glrlm	RunVariance	0.075585149	0.160397408	0.112957612	0.178974972	0.138912221	0.12728559
glrlm	ShortRunEmphasis	-0.10944442	-0.14423928	0.02795521	0.029300805	0.027125111	0.070464131
glrlm	ShortRunHighGrayLevelEmphasis	-0.125807482	0.071837508	-0.031312856	-0.019049437	0.095954661	-0.063155535
glrlm	ShortRunLowGrayLevelEmphasis	0.105477601	-0.036157968	-0.105897725	-0.063135459	0.174092355	-0.132970537

glszm	GrayLevelNonUniformity	-0.105153095	0.085632161	0.039672531	-0.179788464	0.054564393	0.132665388
glszm	GrayLevelNonUniformityNormalized	0.10817899	0.078899517	-0.148280943	-0.083401706	-0.018939871	0.002035075
glszm	GrayLevelVariance	-0.122853455	0.05236549	0.003340686	0.093334572	-0.043586147	-0.161161062
glszm	HighGrayLevelZoneEmphasis	-0.12472589	0.063746685	-0.01290383	0.067476983	0.03593707	-0.145622921
glszm	LargeAreaEmphasis	0.065349342	0.177310116	0.033569226	0.166074428	0.089229462	0.175110901
glszm	LargeAreaHighGrayLevelEmphasis	0.0387698	0.101940463	0.211264044	-0.062147398	0.009419725	0.004616875
glszm	LargeAreaLowGrayLevelEmphasis	0.05691339	0.173186222	-0.03353087	0.188805885	0.091852591	0.209414125
glszm	LowGrayLevelZoneEmphasis	0.11213039	0.036204647	-0.11469107	-0.091794637	-0.006435172	-0.051753122
glszm	SizeZoneNonUniformity	-0.114558961	0.091122742	-0.013945642	-0.122912234	0.130921857	0.009554803
glszm	SizeZoneNonUniformityNormalized	0.045622454	0.120548779	-0.277344352	-0.014408475	-0.035331118	0.005506011
glszm	SmallAreaEmphasis	-0.102113007	-0.053819459	0.054470635	0.059209195	-0.056291146	0.214126331
glszm	SmallAreaHighGrayLevelEmphasis	-0.119464588	0.063626727	-0.028407192	0.091227783	0.037212822	-0.165015145
glszm	SmallAreaLowGrayLevelEmphasis	0.046261233	-0.109484242	0.071411805	-0.073451943	-0.068598431	0.169967862
glszm	ZoneEntropy	-0.125109626	0.007989	0.104829597	0.039489618	-0.039736586	0.043818137
glszm	ZonePercentage	-0.101334431	-0.069085984	-0.120359729	0.125026203	0.143795777	0.097397177
glszm	ZoneVariance	0.042832399	0.101584032	0.216564674	0.212009213	0.209009542	0.150862873
ngtdm	Busyness	0.067726255	0.026688244	0.184642196	0.044267808	0.247258934	-0.003111602
ngtdm	Coarseness	0.058080273	0.157622042	-0.224924288	-0.022147327	-0.125292021	0.029331405
ngtdm	Complexity	-0.118612803	0.061934925	-0.04749836	0.10571901	0.031281478	-0.152607716

ngtdm	Contrast	-0.114901524	-0.04275837	-0.092169754	0.087834874	0.124024506	-0.005675122
ngtdm	Strength	-0.101213785	-0.057167884	-0.066864141	0.238387418	-0.025244277	-0.09110539

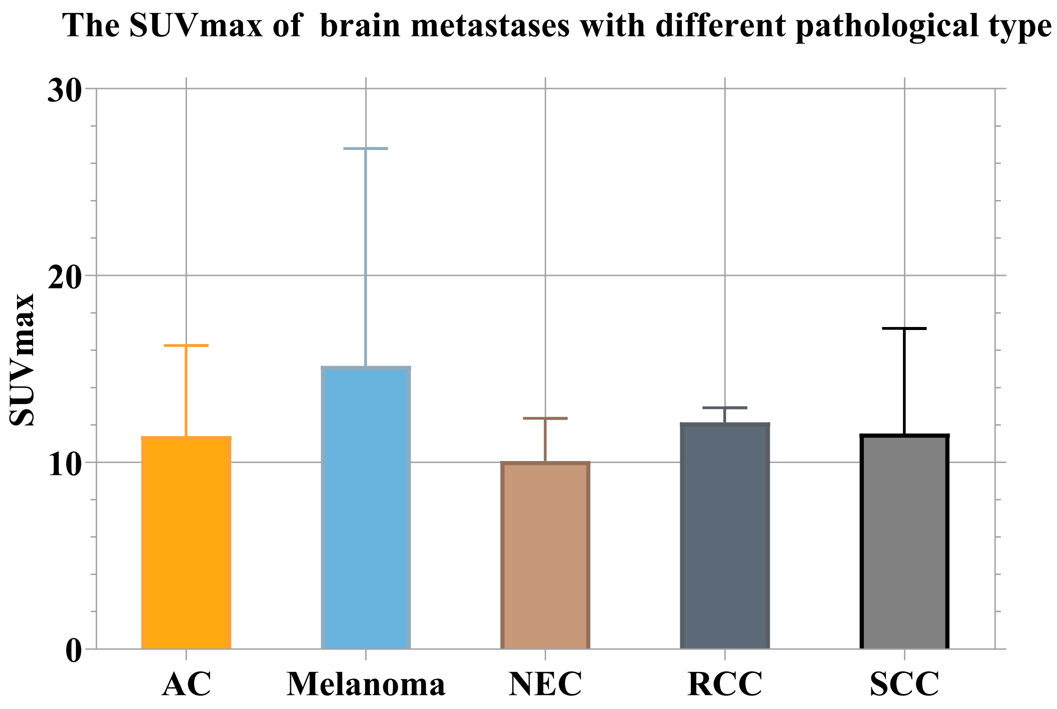

Figure S1. The SUVmax of brain metastases with different pathology.

Figure S1 The average SUVmax values of the lesions with different pathological types. The ANOVA testing found no difference in the average SUVmax among different pathological types (P=0.5213). The average SUVmax of adenocarcinoma, melanoma, neuroendocrine carcinoma, Renal clear cell cancer, and squamous carcinoma was 11.4±4.85, 15.17±11.63, 10.07±2.282, 12.14±0.7797, and 11.54±5.632, respectively.

AC: Adenocarcinoma, NEC: Neuroendocrine carcinoma, SCC: Squamous carcinoma, and RCC: Renal clear cell cancer.
